# Mannitol improves *Monascus* pigment biosynthesis with rice bran as a substrate in *Monascus purpureus*

**DOI:** 10.3389/fmicb.2023.1300461

**Published:** 2023-12-13

**Authors:** Di Chen, Han Li

**Affiliations:** College of Biological Engineering, Henan University of Technology, Zhengzhou, China

**Keywords:** *Monascus* pigments, rice bran, nutritional ingredients, growth and development, transcriptome analysis

## Abstract

To reduce the production cost of *Monascus* pigments (MPs), the utilization of rice bran (RB), an agricultural waste product, as a substrate in submerged fermentation was conducted in this study. To improve MP production, different nutritional ingredients including mannitol (Man), NH_4_NO_3_ (AN), ZnSO_4_ (Zn), and optimization (Opti), which was a synthesis of the three above ones, were added in rice bran (RB) medium. The yields of MPs, pigment constituents, and growth and development of *Monascus purpureus* M9 were investigated in this study. Man had the maximum color value of 3,532 U/g, which was 18.69 times more than that of RB and reached up to 76.65% of the value of rice (Rice) fermentation. Man significantly increased the production of two orange pigments, monascorubrin and rubropunctatin, of which the yields were 69.49 and 95.36% of the counterpart of Rice. The biomass and colony diameter of Opti presented the maximum value among different groups. AN and RB induced more asexual spore formation, whereas Opti and Man promoted sexual spore production. Comparative transcriptomic analysis showed that different nutritional ingredients led to changes in pigment production, promoting the growth and development of *M. purpureus* M9 through the regulation of related gene expression. Man and Opti improved MP production by regulating the primary metabolism, including the Embden–Meyerhof pathway (EMP), the pentose phosphate (PP) pathway, the tricarboxylic (TCA) cycle, fatty acid degradation (FAD), fatty acid biosynthesis (FAB), amino acid metabolism (AAM), and fructose and mannose metabolism (FMM), to provide the precursors (acetyl-CoA and malonyl-CoA) for MP biosynthesis. This study presents a low-cost method for increasing MP production and explains the molecular mechanisms of different nutritional ingredients for enhancing MP biosynthesis.

## Introduction

*Monascus* pigments (MPs), the secondary metabolites produced by the fungal strains of *Monascus* spp., have been used as food colorants for thousands of years in East Asian countries (Feng et al., 2012; Chen et al., 2015). In China, the yields of MPs are nearly 20,000 tons every year. The nationwide market demand for MPs increases progressively with years, with an annual growth rate of 5–10% (Yang et al., 2015). MPs possess several biological properties such as anti-inflammation (Hsu et al., 2012), anti-cancer (Su et al., 2005), anti-microbe (Kim et al., 2006), and anti-obesity (Kim et al., 2007). Besides being used as food colorants, MPs can be potentially used in the pharmaceutical, cosmetic, cotton dyeing, and leather industries (Velmurugan et al., 2009; Lin et al., 2017). MPs are a complex mixture of compounds with a common azaphilone skeleton, which are mainly composed of yellow, orange, and red pigments. To date, at least 100–120 members of MPs have been identified (Guo et al., 2023). Among these MP members, the top six well-known pigments were 2 each of yellow (monascin and ankaflavin), orange (rubropunctatin and monascorubrin), and red (rubropunctamine and monascorubramine) color pigments (Jůzlová et al., 1996). By 2024, the global food colors market is estimated to reach approximately USD$ 5.7 billion (El-Sayed et al., 2022).

MPs have traditionally been produced by the cultivation of *Monascus* strains from rice. However, rice, as the second largest staple food grain, is supplied to half of the world’s population. To reduce the cost of MPs, a new raw material that is readily available and sufficiently cheap will be needed. Rice bran (RB) is generated in large quantities from the industrial process of polishing brown rice to round-grained rice and is generally intended for use as animal feed (Sharif et al., 2014). RB consists mainly of insoluble fiber, protein, fatty acids, and microelement, which can be converted into fermentable ingredients. To encourage sustainable utilization of raw materials, an abundance of agricultural waste products such as jackfruit seeds (Babitha et al., 2007), sugarcane bagasse (Teran Hilares et al., 2018), corn cobs (Velmurugan et al., 2011), potato peels (Srivastav et al., 2015), wheat bran, wheat husks, and rice husks (El-Sayed et al., 2022) have been used for MP production. However, literature concerning the utilization of RB for MP production is scare. Furthermore, the addition of nutritional ingredients can enhance MP production (Huang et al., 2023). In this study, we optimized MP production using different nutritional ingredients with RB.

MP biosynthesis is a complex process governed by pigment biosynthesis of a cluster of genes encoding a series of enzymes such as polyketide synthases, fatty acid synthases, and oxidoreductase. It is not clear that the differential expression level of genes in the whole genome of *Monascus* cultivated with different nutritional ingredients based on RB medium. RNA sequencing (RNA-seq) is a potent and effective way of discovering new genes on a large scale (Liu et al., 2021). It has been widely used to characterize differentially expressed genes (DEGs) under various conditions (Shin et al., 2016).

To improve MP production, different nutritional ingredients, including mannitol (Man), NH_4_NO_3_ (AN), ZnSO_4_ (Zn), and optimization (Opti), were added in RB medium. The yields of MPs, pigment constituents, and growth and development of *M. purpureus* M9 were investigated in this study. Furthermore, transcriptome analyses were performed to explore DEGs of *M. purpureus* M9, which helped explain the molecular mechanisms of MP biosynthesis.

## Materials and methods

### Microorganism and culture conditions

*Monascus purpureus* M9 (strain NO. CGMCC 3.19586) was maintained on malt extract agar slants with a sugar content of 10°Bx. The slant was washed with 3 mL distilled water, which was transferred to the culture medium (glucose, 60 g/L; peptone, 20 g/L; KH_2_PO_4_, 10 g/L; NaNO_3_, 10 g/L; and MgSO_4_, 5 g/L; pH 4.5). The culture medium was placed in a rotary HZQ-C shaker (HDL, Harbin, China) at 180 rpm and 28°C for 36 h. Spore suspension was obtained by filtering the aforementioned inoculum with 8-layer sterile gauze, and the concentration of spore suspension was adjusted to 10^6^ spores/ml.

For pigment fermentation, 3 mL of spore suspension was inoculated into 50 mL of RB medium in a 250-ml flask. Cultures were incubated at 28°C and 180 rpm for 7 days. Six kinds of submerged fermentation culture media were prepared as follows: (1) rice bran, 50 g/L, (2) rice bran, 50 g/L and mannitol, 50 g/L, (3) rice bran, 50 g/L and NH_4_NO_3_, 10 g/L, (4) rice bran, 50 g/L and ZnSO_4_, 4 g/L, (5) rice bran, 50 g/L; mannitol, 50 g/L; NH_4_NO_3_, 10 g/L; and ZnSO_4_, 4 g/L, (6) rice powder, 50 g/L. To the corresponding solid media, 25 g/L of extra agar powder were added.

### Measurement of biomass and colony diameter

At the center of solid medium, 10 μL of spore suspension (10^6^ spores/mL) was added, and the surface of the suspension was covered with cellophane membranes. The colonies were cultivated for 9 days at 28°C. The colony diameter was measured using a vernier caliper from 3 days to 9 days. Meanwhile, the weights of fresh colony uncovered from cellophane membranes were determined using the electronic scale AUY120 Analytical Balance 120 g Capacity (Shimadzu, Japan).

### Development analysis

To quantify cleistothecia and conidia, a cork borer with a diameter of 1 cm was used to excise agar cylinders from the plate and placed in a test tube with 1 mL of sterile water and was vortexed to dislodge conidia and cleistothecia, which were counted using a hemacytometer.

### Morphological evaluation

To observe the colony phenotype and microscopic morphology, six kinds of solid media inoculated with 5 μL of a conidial suspension (10^5^ spores/mL) were incubated at 28°C for 9 days. The spores and fresh aerial hyphae were collected from plates and observed through an optical microscope Eclipse E200 (Nikon, Japan). The change in colony color was recorded by an IXUS 105 camera (Canon, Japan).

### Color value determination

Freshly harvested mycelia were taken from the fermentation medium on the 7th day to measure intracellular MP productions. Then, they were dried and ground to dust. Later, dried mycelia (0.5 g) were extracted with 75% ethanol (3 mL) with sonication of 30 min and then centrifuged at 2,862 × g for 10 min to collect the supernatant. The experiment was conducted in triplicate. All supernatants were merged for the determination of intracellular pigmentation. The solution from pigment extraction was determined by a Cary-3500 UV/VIS spectrophotometer (Agilent, California, United States) at a specific wavelength of 505 nm. The absorbance at the λ_max_ of the pigment per gram of dry mycelia was used to express the color value.

### LC–MS and HPLC analysis of MPs

The abovementioned solution from pigment extraction were analyzed qualitatively by Waters XEVO-TQD QCA1534 LC/MS System (Waters, Massachusetts, United States) equipped with an electrospray ionization (ESI) source in positive and negative models. Full scans were performed between m/z values 300 and 500. The electrospray conditions were as follows: capillary voltage, 3.5 kV; nebulizer pressure, 35 psi; drying gas flow, 10.0 mL/min; and temperature, 350°C. A total of 10 μL of the sample was added for LC–MS analysis with a chromatographic column (C18, 150 × 3.0 mm, 2.6 μm, Phenomenex, California, United States). The eluent and elution program were the same as HPLC. The ion chromatogram of a single component was extracted and referred them with parent ion and daughter ion values as follows: monascorubramine (parent ion: m/z 354.2; daughter ion: m/z 310.1, 292.1) and rubropunctamine (382.2; 179.1, 160.1), monascin (359.3; 287.3, 215.1) and ankaflavin (387.3; 311.2, 261.1), and monascorubrin (355.2, 311.2, 293.1) and rubropunctatin (383.2, 339.2, 321.2).

The quantitative analysis of six main MPs was performed by 1,200 HPLC system (Agilent, California, United States) coupled with a diode array detector (DAD). Pigments were separated by a reverse-phase column (XDB C18, 150 × 4.6 mm, 5 μm, Agilent, California, United States) with a flow rate of 1.0 mL/min. The mobile phases were solvent A (0.1% formic acid in water) and solvent B (acetonitrile). A gradient elution was performed as follows: solvent B was maintained at 60% for 12 min, 60 to 90% for 13 min, 90% for 2 min, 90 to 60% for 3 min. The detection wavelength was 410 nm. A volume of 20 μL of the sample was used for each experiment.

### Detection of rice and rice bran composition

Amylose, amylopectin, protein, and fat content of Rice and RB were determined according to the method of Cheng et al. (2022). For starch assay, samples were dispersed using 1 mol/L of KOH solution and heated in a boiling water bath (100°C) for 10 min. Subsequently, the content was cooled, and the sample solution was filled in a 100-ml flask. Then, approximately 2.5 mL of this prepared sample solution was added with 1 mL of HCL (0.1 mol/L) and 2 mL of iodine solution to observe color reaction. The absorbance was measured at 620 nm with a spectrophotometer (Agilent, California, United States). The standard curves of amylose and amylopectin were plotted using standard substances. For crude protein assay, samples were digested by H_2_SO_4_ at 420°C for 1 h. The crude protein content was estimated using the Kjeldahl method. The fat content of the samples was detected using the Soxhlet extraction method.

Mineral elements of Rice and RB were evaluated using methods described by da Silva et al. (2013). Then, 0.5 g of the samples was weighed accurately and poured into the digestion vessel, followed by the addition of 10 mL HNO_3_. The digestion vessels were capped and heated in the CEM MARS5 Microwave Accelerated Reaction System (CEM Corporation, North Carolina, United States). After digestion, the solutions were cooled to room temperature and then filled into a 25-ml flask. Three samples including a digestion reagent with no samples, a standard reference material, and experimental samples were prepared. Inductively Coupled Plasma Mass Spectrometry (ICP-MS; Agilent, California, United States) was used to quantify the mineral element content.

### Comparative transcriptomic analysis

Mycelia cultured on the 7th day for RB, Man, AN, Opti and Rice were rapidly frozen using liquid nitrogen and immediately transferred to a -80°C freezer for storage. They were submitted to the BGI Group (Shenzhen, China) for RNA extraction, cDNA library construction, and RNA-seq analysis. Total RNA of *M. purpureus* M9 mycelia was extracted according to the manufacturer’s protocol. The RNA concentration and integrity were measured following the method described by Jian et al. (2018). Sequencing libraries were constructed using the NEBNext Ultra™ RNA Library Prep Kit for Illumina HiSeq 4,000 systems (California, United States). Clean data were obtained by removing the adapter, sequencing primers, and low-quality reads. The sequencing error distribution rate check (Q20 and Q30) was calculated to verify the quality of the sequencing. Fragments per kilobase of transcript per million mapped reads (FPKM) were calculated to estimate the gene expression level of the samples. Three biological replicates were used for RNA-seq analyses.

For RNA-seq bioinformatic analysis, the DESeq2 package was used for the calculation of differential expression analysis. DEGs were screened according to the baseline of *p*-value ≤0.01 and |log2 fold change| ≥2. DEG functions were annotated to the Gene Ontology database (GO) using BLAST software. The metabolic pathways and functions of genes were systematically analyzed using the Kyoto Encyclopedia of Genes and Genomes (KEGG) database. KOBAS 2.0 was used to calculate the statistical enrichment of DEGs in KEGG pathways (Xie et al., 2011).

### Validation of the selected genes expression level by RT-qPCR

The gene expression change was verified by real-time quantitative polymerase chain reaction (RT-qPCR). Then, total RNA of the mycelia was extracted by the plant RNA Kit (Omega Bio-tek, California, United States) and reverse transcribed using the PrimeScript^™^ RT reagent kit with genomic DNA Eraser (TaKaRa, Tokyo, Japan) for complementary DNA. The expression of the selected genes was determined using SYBR Premix Ex Taq II (TaKaRa Tokyo, Japan) by RT-qPCR using the Stratagen Mx3000P (Agilent, California, United States). The β-actin gene was used as a reference gene. All primers used in this study were listed in Supplementary Table S1.

### Statistical analysis

Each experiment was performed in triplicate, and the results were expressed as the mean ± standard deviation. Statistical significance was determined by a one-way analysis of variance (ANOVA) with SPSS 17.0 software. *p*-values of <0.05 and < 0.01 were considered statistically significant.

## Results

### Effects of different nutritional ingredients on MP production

**Figure 1:**
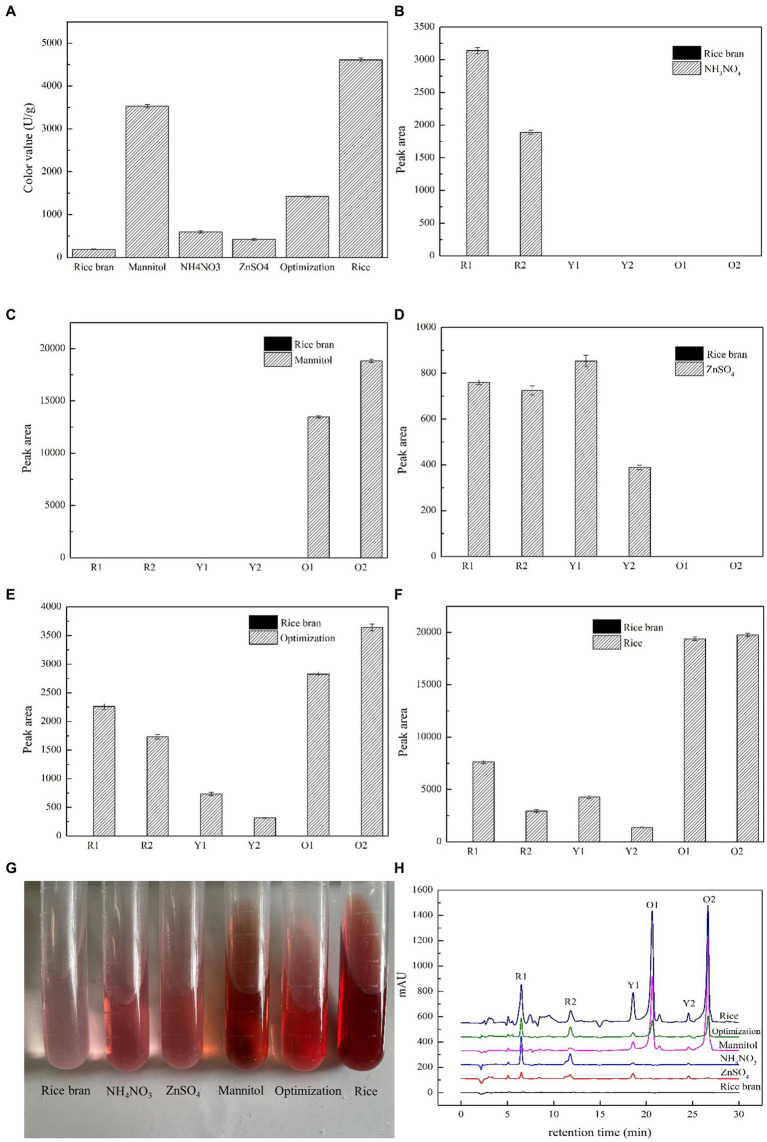
Effects of nutritional ingredients on the concentration and constituent of MPs in *M. purpureus* M9. The concentration of MPs was assessed by the absorbance value of 505 nm. The color values were expressed as OD units per gram of dry mycelia **(A)**. Yields of the six classical MPs were assessed by relative peak areas provided by HPLC under different conditions in which the rice bran substrate was added with NH_4_NO_3_ **(B)**, mannitol **(C)**, ZnSO_4_ **(D)**, and optimization **(E)** and mixed with the three above ingredients. Rice medium **(F)** was used as the control group. The images of intracellular pigments extracts **(G)** and the HPLC profiles **(H)** were both exhibited.

In our previous study, a series of nutritional ingredients, such as carbon source, nitrogen source, and mineral salt ion, were added individually in RB medium to enhance MP biosynthesis. As a result, Man, AN, and Zn, respectively, were the most efficient carbon source, nitrogen source, and mineral salt ion for MP production. In Figure 1A, the color values for AN and Zn were approximately the same, with Man having the maximum color value of 3,532 U/g, which was 18.69 times more than that of RB and reached up to 76.65% of the value of Rice. Interestingly, the yields of Opti, mixed with Man, AN, and Zn, were 1,424 U/g, which was lower than Man. These results showed that carbon source, such as Man, might act as a major player in MP production based on RB medium.

The above findings were confirmed by the composition of RB and Rice (Table 1). Although the total starch, amylopectin, and amylose contents of RB were much less than that of Rice, the fat and protein concentrations of RB were significantly higher than that of Rice. In addition, rich mineral salt ions were detected in RB, for instance Mg, Mn, Fe, Cu, and Zn. In RB, Mg content reached 2,092.15 mg/kg, which was 3.08-fold higher than that of Rice. In addition, Mn, Fe, Cu, and Zn contents exhibited similar trends. As shown in Table 1, the composition of fat, protein, and mineral contents of RB was much higher than that of Rice, while starch content composition was less than that of Rice. Therefore, the addition of carbon source had significant effects on MP biosynthesis based on RB medium.

**Table 1:** Nutritional components of rice bran and rice.

Variety	Total Starch content (%)	Amylose content (%)	Amylopectin content (%)	Crude fat content (%)	Protein content (%)	Mg (mg/Kg)	Mn (mg/Kg)	Fe (mg/Kg)	Cu (mg/Kg)	Zn (mg/Kg)
Rice bran	12.74 ± 0.14^b^	1.51 ± 0.06^b^	11.23 ± 0.08^b^	23.41 ± 0.23^a^	13.11 ± 0.09^a^	2092.15 ± 0.33^a^	327.70 ± 0.21^a^	118.34 ± 0.13^a^	7.24 ± 0.07^a^	43.84 ± 0.10^a^
Rice	68.01 ± 0.17^a^	9.74 ± 0.06a	58.27 ± 0.11^a^	8.16 ± 0.08^b^	7.56 ± 0.07^b^	679.16 ± 0.25^b^	25.22 ± 0.11^b^	29.20 ± 0.12^b^	1.99 ± 0.03^b^	17.69 ± 0.07^b^

Data with different letters in the same column were significantly different (*p* < 0.05).

### Different nutritional ingredients transformed the six classical MP production by HPLC

The color of MPs was changed by the addition of different nutritional ingredients (Figure 1G). The color characteristics of MPs were largely dependent on the component concentration in the mixture. Therefore, the yields of six major compounds of MPs, namely yellow pigments of monascin (Y1) and ankaflavin (Y2), orange pigments of monascorubrin (O1) and rubropunctatin (O2), and red pigments of monascorubramine (R1) and rubropunctamine (R2), were analyzed by HPLC. The six classic MPs were identified by LC–MS and spectral analysis (Supplementary Figure S1). The main approaches for distinguishing MPs were based on the m/z value provided by LC–MS, spectrogram of DAD, and retention time. For example, in the Man group, the retention times of O1 and O2 were 20.64 min and 26.66 min. Their m/z values were 355.3 and 383.3, respectively, which were the same as the m/z value (in [M + H]^+^ mode) of monascorubrin and rubropunctatin. The spectrograms of monascorubrin and rubropunctatin were consistent with the characteristics of orange pigments (Teng and Feldheim, 1998). Thus, O1 and O2 were confirmed as monascorubrin and rubropunctatin.

In Figure 1, the addition of AN in RB culture medium produced two red pigments, R1 and R2. Meanwhile, two orange pigments, O1 and O2, were emerged with the addition of Man, of which the yields were significantly higher than that of other groups. The utilization of Zn generated a small quantity of red pigments and yellow pigments. However, the Opti group produced six pigments, R1 and R2, Y1 and Y2, and O1 and O2, all of which were the same as that produced by the Rice group. When only RB was used as culture medium, the amounts of six MPs were greatly lowered to the undetectable level. There were obvious differences on the HPLC profiles between different nutritional ingredients groups (Figure 1H). The peak pattern changes were consistent with the variation in peak areas.

### Effects of different nutritional ingredients on fungal phenotype

**Figure 2:**
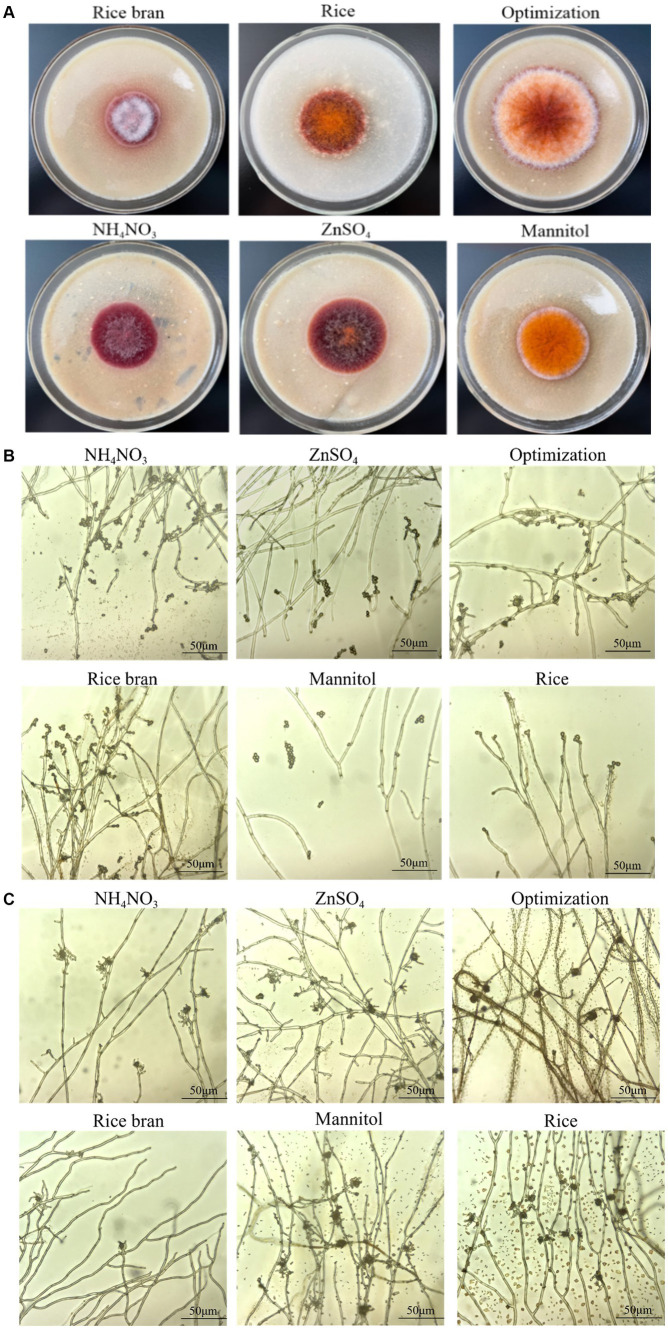
Colony phenotype and microscopic morphology of *M. purpureus* M9 grown with different nutritional ingredients. Colony phenotype of strains grown on rice bran, NH_4_NO_3_, optimization, ZnSO_4_, mannitol, and rice media at 28°C on the 9th day was photographed **(A)**. The asexual **(B)** and sexual **(C)** developments of strain grown on the above media were observed via an optical microscope.

In terms of the growth and morphology of *M. purpureus* M9, different nutritional ingredients produced significant differences. These colonies were cultivated to the same day (the 9th day), but the colony size was obviously different (Figure 2A). The colony diameter of the Opti group was significantly larger than that of others, whereas the minimum value was significantly higher in the RB group than that of others. These colonies were felted, with many mycelia. The hyphae of the Man, Opti, and Rice groups were dense, while sparse mycelia were observed in those of the AN and Zn groups. Moreover, the color characteristics exhibited by the colonies was very distinct. In brief, the Man sample presented in bright orange, and the Opti and Rice groups presented in orangish red. The AN and Zn group samples presented in deep red, while white colony was observed in the RB group. These results coincided with pigment constituent production.

The microcosmic morphologies of mycelia with the addition of different nutritional ingredients are shown in Figures 2B,C. The hyphae were transverse septa, with them being multinucleate, branching, and irregular. The conidia were attached to the tips of the mycelium and its branches, while the closed capsule shells were spherical. Significant differences in conidia and cleistothecia formation were observed among different nutritional ingredients groups. The numbers of conidia in the AN and RB groups were noticeably higher than that of others. It is well known that disadvantaged circumstances easily lead to conidia formation. The results suggested that it is difficult to maintain *M. purpureus* M9 growth and development with the utilization of only RB or the addition of AN. In contrast, cleistothecia in the Man and Opti groups were full, smooth, hydrated, and dense, which presented the similar state with the Rice group, whereas those in the AN and RB groups became irregular and sparsely distributed.

### Effects of different nutritional ingredients on fungal growth and development

**Figure 3:**
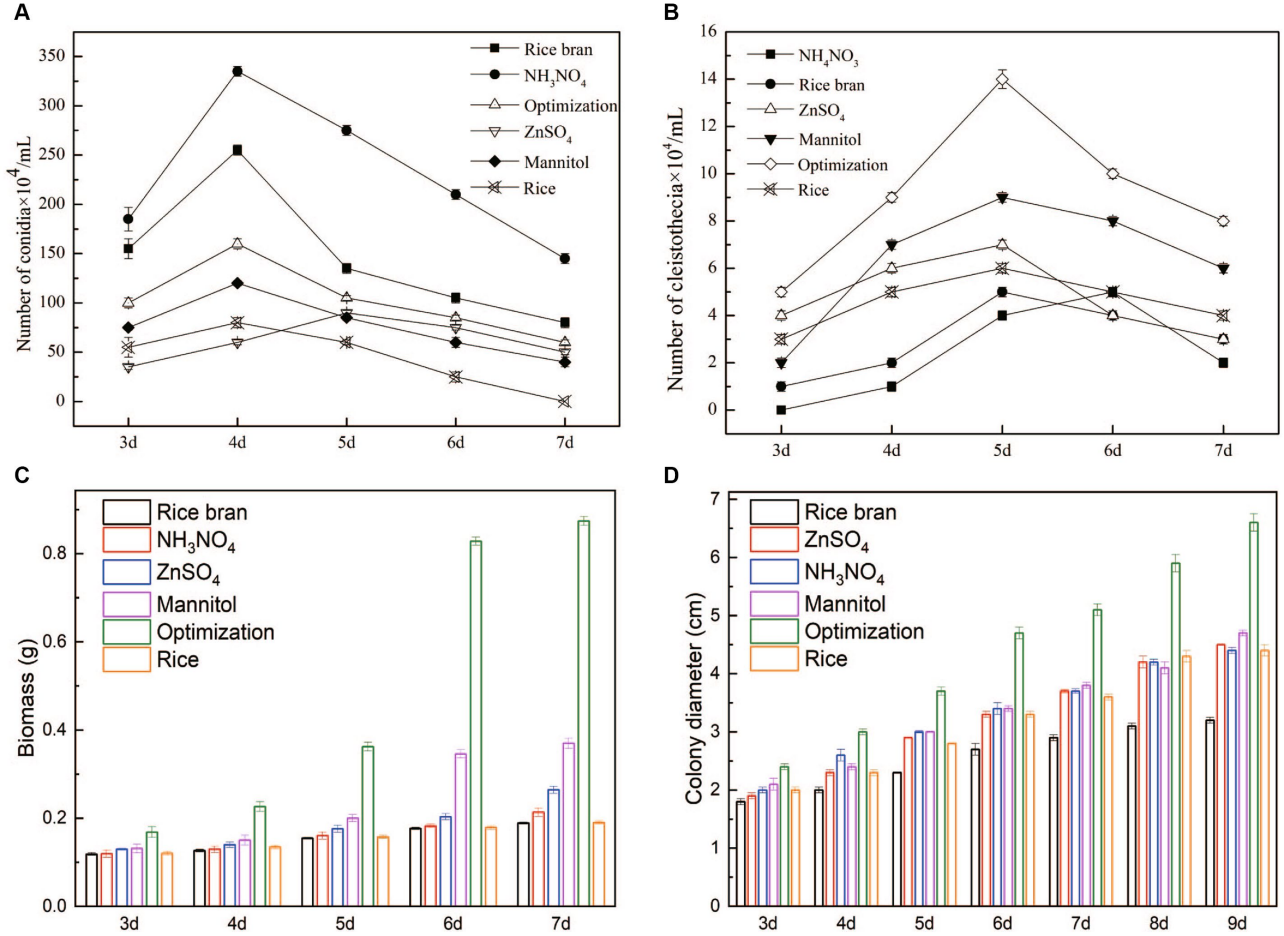
Effects of nutritional ingredients on growth and development in *M. purpureus* M9. Strains were grown on rice bran, NH_4_NO_3_, optimization, ZnSO_4_, mannitol, and rice media at 28°C from 3 days to 9 days. The conidia **(A)** and cleistothecia **(B)** were determined by a hemacytometer. Biomass was weighed from 3 days to 7 days **(C)**, and the colony diameter was measured using a vernier caliper from 3 days to 9 days **(D)**.

*Monascus* spp. goes through two developmental phases during its life cycle: a sexual and an asexual one, which led to the formation of cleistothecia and conidia, respectively (Etxebeste et al., 2019). With the extension of fermentation time, the number of conidia was increased at first and then decreased. The maximum amount of conidia was obtained on the 4th day of fermentation. Conidia productions of the AN and RB groups were significantly higher than that of the others (Figure 3A). In contrast, the amount of cleistothecia were gradually increased and reached the maximum value on the 5th day of fermentation. The order of cleistothecia yields was Opti>Man>Zn>Rice>RB>AN (Figure 3B). The results suggested that Opti and Man were more favorable to sexual spore generation, while AN and RB was conducive to produce asexual spores.

With respect to biomass, the biomass was gradually increased with an increase in fermentation time. The Opti group was much greater than that of others, indicating that the synthesis of carbon source, nitrogen source, and mineral ion source was important for hyphal growth in *Monascus* fungi (Figure 3C). The colony diameter exhibited similar trends with biomass (Figure 3D).

### RNA-seq and read assembly of *Monascus purpureus* M9 cultivated with different nutritional ingredients

The Zn group phenotype was similar to AN, thus RNA-seq was performed with five kinds of nutritional ingredients: RB, AN, Man, Opti, and Rice. After fastp support filtering, each sample produced 42.02–42.76 million clean reads and 6.3–6.41 billion clean base pairs (Supplementary Table S2). The mean values of Q20 and Q30 were greater than 94 and 88%, respectively. Using HISAT2 software, approximately 85.22% of the clean reads for each sample were uniquely mapped to the *M. purpureus* reference genome,^1^ suggesting from the transcriptome profiles that RNA-seq quality was good in this study (Supplementary Table S3).

The Spearman correlation coefficient analysis showed that the biological replicates ranged from 0.98 ~ 0.99, demonstrating a high degree of correlation between them (Supplementary Figure S2). Principal component analysis (PCA) is a linear transformation that reduces high-dimensional data to two or three dimensions. PCA analysis can cluster similar samples together and the closer the distance, the higher the similarity between samples (Xie et al., 2022). The transcriptional program of *M. purpureus* M9 differed significantly in thee RB vs. AN, RB vs. Rice, RB vs. Man, and RB vs. Opti groups. However, Man and Opti were more similar, indicating a high similarity in their transcriptional patterns (Supplementary Figure S3). The expression level of each of the 7,726 genes was determined in this study. A total of 4,388, 4,569, 5,283, and 4,854 DEGs were identified between the AN vs. RB, Man vs. RB, Opti vs. RB, and Rice vs. RB groups, respectively.

### GO functional classification and KEGG pathway enrichment of differentially expressed genes

**Figure 4:**
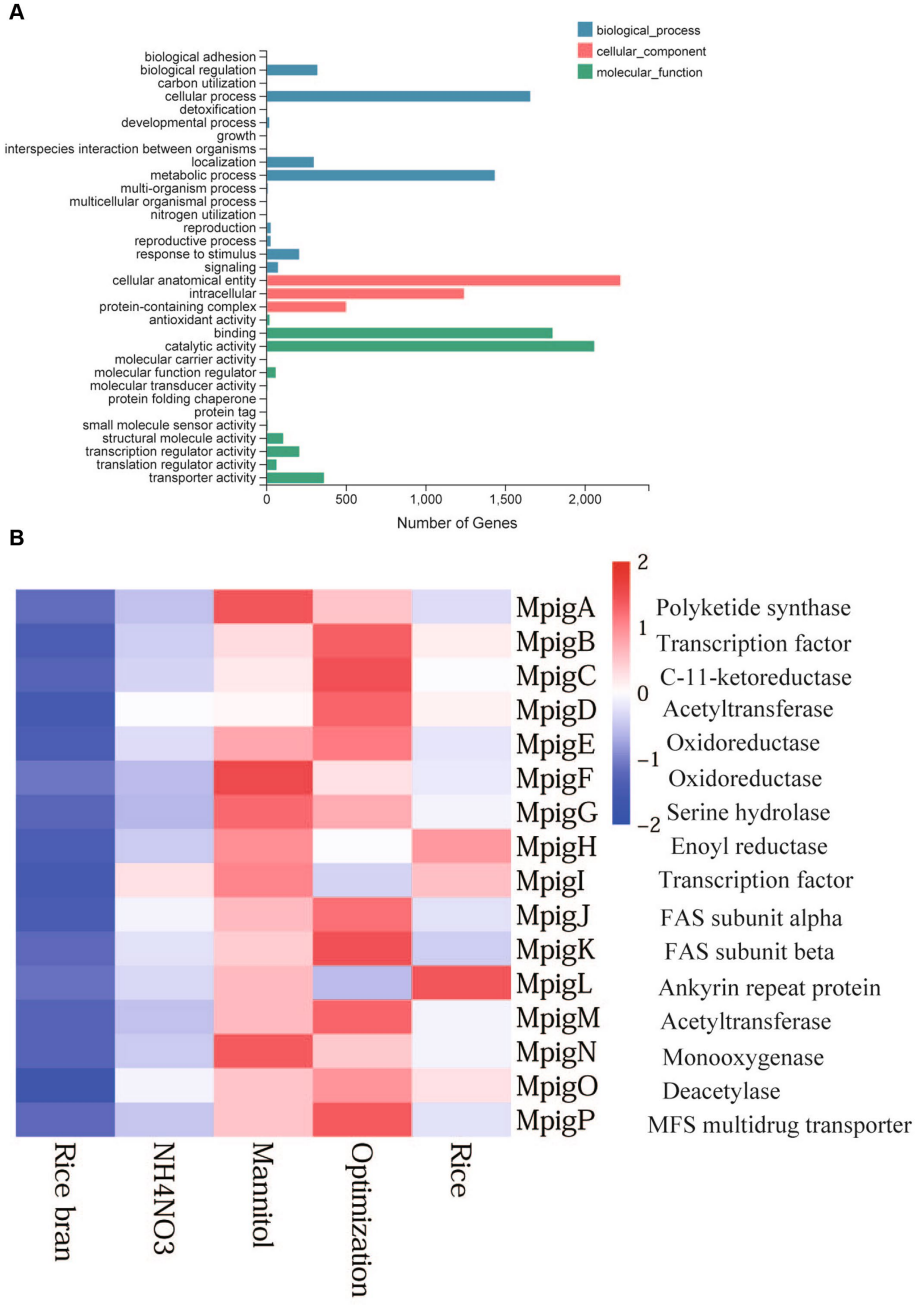
GO function classification **(A)** and expression profiles of genes involved in MP biosynthetic clusters **(B)**.

The results of GO classification were classified as three major classes: biological processes, molecular function, and cellular composition. The biological process groups were subdivided into cellular process, metabolic process, biological regulation, developmental process, localization, reproduction, reproductive process, and response to stimulus. The subdivision for molecular function led to catalytic activity, binding, molecular function regulator, transcription regulator activity, translation regulator activity, and transporter activity. In terms of cellular components, the top three GO items were cellular anatomical entity, intracellular, and protein- containing complex. These observations showed that significantly enrichment GO term mainly concentrated on development and reproduction (Figure 4A).

**Figure 5:**
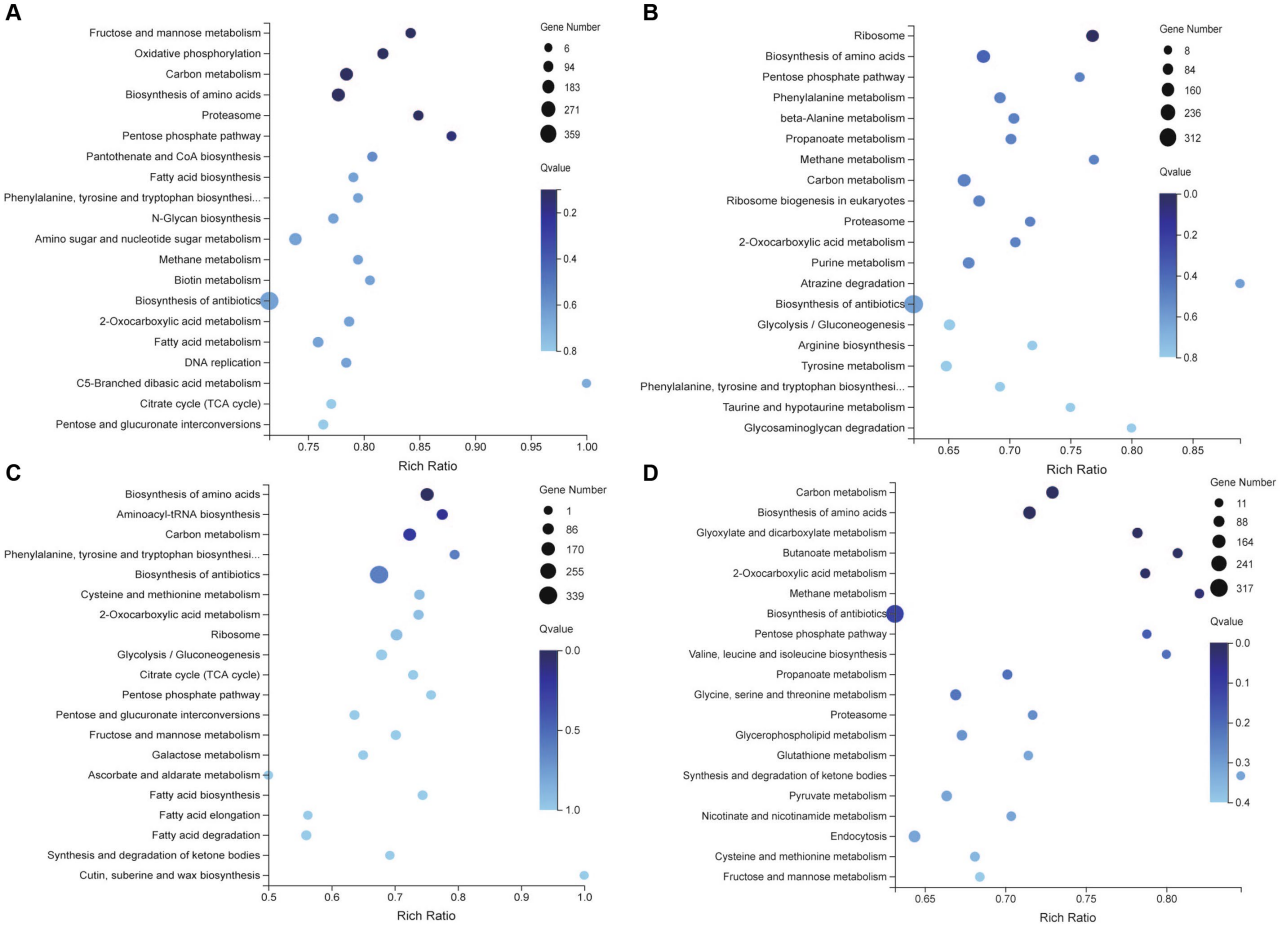
KEGG enrichment analysis of differentially expressed genes involving Opti vs. RB **(A)**, Man vs. RB **(B)**, Rice vs. RB **(C)**, and AN vs. RB **(D)** groups.

KEGG pathway analysis revealed that DEGs were significantly enriched in the metabolism and biosynthesis pathways. Compared with the RB group, Opti, Man, and Rice were all enriched in tryptophan, phenylalanine, and tyrosine biosynthesis pathways (Figures 5A–C). Tryptophan, phenylalanine, and tyrosine are aromatic amino acids and can be converted into several biological compounds by a series of oxidation reactions, including 5-hydroxy tryptamine, heteroauxin, dopamine, and melanin involvement in the physiology and growth of organisms (Chen D. et al., 2017), which suggested that Opti, Man, and Rice significantly affected the growth and development of *M. purpureus* M9. Glycolysis/gluconeogenesis, citrate cycle, pentose phosphate (PP) pathway, and fatty acid metabolism were enrichment pathways exhibited in the Man vs. RB, Opti vs. RB, and Rice vs. RB groups (Figures 5A–C). Glycolysis, occurring in the cytoplasm of almost all organisms, can be defined as a metabolic pathway of glucose to produce acetyl-CoA and malonyl-CoA, which are important precursors for the biosynthesis of MPs (Guo et al., 2023). Therefore, the Man, Opti, and Rice groups regulated the primary metabolism for MP production.

During MP biosynthesis, the classical red MPs, R1and R2, originate by the direct reaction of O1 and O2 with endogenous amines, especially amino acids (Chen W. et al., 2017). In the AN vs. RB group, DEGs were significantly distributed in amino acid metabolism, such as valine, leucine, and isoleucine biosynthesis, glycine, serine and threonine metabolism, and cysteine and methionine metabolism (Figure 5D). Most of these metabolites are amine derivatives, which might be the reason for accumulates R1 and R2 in the AN group.

### Expression level analysis of genes related to pigment biosynthesis

The gene cluster of MP biosynthesis consists of a series of highly conserved genes, with 12 genes encoding enzymes associated with MP biosynthesis, and the remaining genes were involved in transportation and regulatory factors (Figure 4B). To investigate expression profile of these genes under different nutritional ingredient conditions, a heat map was created based on the total RNA expression of five samples. The colored scale varies from deep blue to deep red, which indicates low or high expression. The heat map shows that the expression levels of genes in the RB and AN groups were low, while the Opti, Man, and Rice groups showed higher transcription levels. The results were consistent with the changes in pigment production. In pigment production section, the color value of Rice, Man and Opti were noticeably higher than that of AN and RB. Man significantly improved MP production. Accordingly, the expression levels of genes in Man were the highest among the AN, Opti, and Man groups.

### Analysis of differentially expressed genes involved in growth and reproduction

DEGs related to sexual and asexual development of *M. purpureus* M9 cultivated with different nutritional ingredients were investigated in this study. Among 27 DEGs related to development, the expression levels of 8 genes linked to asexual sporulation were upregulated in the AN vs. RB group but downregulated in the Opti vs. RB and Man vs. RB groups (Table 2). These genes include Heterokaryon incompatibility protein 6, OR allele involved in asexual growth (Smith et al., 2000); Conidiation-specific protein 8, which contributes to conidium formation; Cell wall protein PhiA and Chitin synthase D required for conidium cell wall organization; and Protein SUR7 related to spore release in response to stress. In terms of sexual development, several genes associated with sexual development were upregulated in the Man vs. RB, Opti vs. RB, and Rice vs. RB groups, whereas these genes trended to be downregulated or not significantly changed in the AN vs. RB group, such as Pheromone P-factor receptor, which is involved in the initiation of meiosis (Wood et al., 2002), Meiotically up-regulated gene 70 protein, which plays a role in meiosis (Ding et al., 2000), and Rho guanine nucleotide exchange factor scd1, Serine protease EDA2, 1,3-beta-glucanosyltransferase gas2, Sexual differentiation process protein isp4, and Mating-type protein MAT-1, which participate in spore wall assembly (Pagnussat et al., 2005). The results were consistent with the phenotypic observation that AN induced more conidia formation, whereas Man and Opti promoted more sexual spore production. Moreover, some genes associated with fungal growth were upregulated in the Opti vs. RB groups included Serine/threonine-protein kinase mph1, which is associated with initiating mitosis (He et al., 1998); Serine/threonine-protein kinase ark1, Kinesin-like protein KIF22, and WD repeat-containing protein slp1, which are involved in spindle formation (Huang et al., 2005); and Checkpoint serine/threonine-protein kinase bub1and G2/mitotic-specific cyclin-B, which are linked to the spindle formation checkpoint (Kadura et al., 2005), suggesting that the Opti group facilitated growth and reproduction in *M. purpureus* M9. This result was in line with the biomass and colony diameter data.

**Table 2:** Differentially expressed genes related to reproduction and development.

Protein identification	log_2_FoldChange				Involvement processes
	AN vs. RB	MAN vs. RB	Opti vs. RB	Rice vs. RB	
Sexual process					
Sexual differentiation process protein isp4	NS	Up	Up	Up	Sexual differentiation process
Mating-type protein MAT-1	NS	Up	NS	Up	Sexual differentiation process
1,3-beta-glucanosyltransferase gas2	NS	Up	Up	NS	Involved in spore wall assembly
Serine protease EDA2	NS	NS	Up	Up	Gametgenesis
Meiotically up-regulated gene 70 protein	Down	Up	NS	Up	meiosis
Pheromone P-factor receptor	Down	Up	NS	Up	Initiation of meiosis
Rho guanine nucleotide exchange factor scd1	NS	Up	Up	Up	Mating and morphogenesis
Asexual process					
Cell wall protein PhiA	Up	Down	NS	NS	Cell wall of asexual spore
glucan 1,3-beta-glucosidase A	Up	Down	Down	NS	Spore formation and release
Chitin synthase D	Up	Down	Down	Down	Cell wall biosynthetic process
Endo-1,6-alpha-mannosidase DCW1	Up	Down	Down	Down	Cell wall biosynthetic process
Incompatibility protein 6, OR allele	Up	NS	-	NS	Asexual reproduction
Protein SUR7	NS	Down	Down	Down	Spore formation
Meiotically up-regulated gene 14 protein	NS	Down	Down	NS	Mitosis
Conidiation-specific protein-8	Up	Down	Down	Up	Asexual spore formation
Development process					
Meiotic recombination protein SPO11	NS	Up	NS	Up	Asexual and Sexual
Serine/threonine-protein kinase ark1	NS	Up	Up	NS	Spindle formation and chromosomal alignment
Checkpoint serine/threonine-protein kinase bub1	NS	NS	Up	Up	Spindle-assembly checkpoint and chromosomal alignment
Serine/threonine-protein kinase mph1	Down	NS	Up	Down	Initiating mitosis
G2/mitotic-specific cyclin-B	Down	Up	Up	Down	Cell cycle at the G2/M (mitosis) transition
Metallothionein expression activator	NS	Up	Up	NS	The transcription of genes required for cell separation
G2/mitotic-specific cyclin-4	NS	Down	NS	Down	Cell cycle at the G2/M (mitosis) transition
Glucan endo-1,3-alpha-glucosidase agn1	NS	Up	Up	NS	The degradation of the cell wall material
Kinesin-like protein KIF22	NS	NS	Up	NS	Spindle formation and the movements of chromosomes during mitosis and meiosis
Separin	NS	Down	Up	Down	Chromosome segregation
G2-specific protein kinase nimA	NS	NS	Up	NS	Spindle formation and the movements of chromosomes during mitosis and meiosis
WD repeat-containing protein slp1	NS	Up	Up	NS	Spindle formation

Up and Down mean the metabolites up- and down-regulated in the gene transcriptional level; NS represented not significantly changed; “-” was not detected.

### Effects of different nutritional ingredients on the primary metabolism

According to Table 3, different nutritional ingredients exerted a global influence on the primary metabolisms, including Embden–Meyerhof pathway (EMP), tricarboxylic acid (TCA) cycle, pentose phosphate (PP) pathway, fatty acid degradation (FAD), amino acid metabolism (AAM), and fructose and mannose metabolism (FMM). Compared with the RB group, the Man, Opti, Rice, and AN groups had positive effects on the transcriptional levels of genes encoding enzymes involved in EMP, such as glucose-6-phosphate isomerase, phosphoglycerate mutase, 6-phosphofructokinase, enolase, phosphoglycerate kinase, pyruvate kinase, pyruvate dehydrogenase, and glyceraldehyde-3-phosphate dehydrogenase, which led to higher concentrations of precursors (acetyl-CoA and malonyl-CoA) for MP biosynthesis (Huang et al., 2018), but genes encoding hexokinase were downregulated. Furthermore, the TCA cycle was also affected by different nutritional ingredients, which were associated with the higher transcriptional level of citrate synthase, isocitrate dehydrogenase, malate dehydrogenase, succinate dehydrogenase, and alpha-ketoglutarate dehydrogenase complex. While the expression levels of genes encoding succinyl-CoA synthetase and aconitate hydratase were downregulated, the expressing levels of genes encoding transketolase and 6-phosphogluconolactonase involved in the PP pathway were upregulated, while ribose 5-phosphate isomerase A showed a lower transcriptional level. The PP pathway is closely associated with EMP and could further affect carton flux toward acetyl-CoA and malonyl-CoA by EMP. Importantly, nicotinamide adenine dinucleotide phosphate (NADPH) can be biosynthesized in the PP pathway, which can provide a reducing agent for the biosynthesis of pigments (Hong et al., 2020). Moreover, the metabolic processes of fatty acids are important for MP production (Wang et al., 2015). The transcriptional levels of genes in the fatty acid biosynthesis (FAB) pathway, such as genes encoding fatty acid synthase beta subunit, fatty acid synthase alpha subunit FasA, and palmitoyl-protein thioesterase were downregulated, while the transcriptional levels of genes in the FAD pathway to acetyl-CoA by beta-oxidation was accelerated, which manifested as a result of increased transcription levels of genes encoding enoyl-CoA hydratase and acyl-CoA dehydrogenase. Thus, the pathways of upregulation of FAD and downregulation of FAB could offer more substrates for MP biosynthesis. In addition, amino acids play a positive role in producing intermediates (pyruvate, alpha-ketoglutarate, or oxalacetate) for the TCA cycle and further influence MP biosynthesis (Huang et al., 2021). The transcriptional levels of genes involved in AAM, namely glycine hydroxymethyltransferase, ketol-acid reductoisomerase, chorismate mutase, anthranilate phosphoribosyltransferase, and O-acetylhomoserine, were upregulated, whereas 3-isopropylmalate dehydratase, NAD^+^ dependent glutamate dehydrogenase, and aspartate aminotransferase were downregulated, indicating different nutritional ingredients that influenced the conversion of amino acids into the intermediates for the TCA cycle. Besides, Man significantly improved MP production, thus FMM were concerned in this study. Mannitol-1-phosphate 5-dehydrogenase and mannitol 2-dehydrogenase were notably upregulated in the Man group.

**Table 3:** Differentially expressed genes related to primary metabolism.

Protein identification	log_2_FoldChange			
	AN vs. RB	MAN vs. RB	Opti vs. RB	Rice vs. RB
Glycolysis / Gluconeogenesis (EMP)				
Glucose-6-phosphate isomerase	Up	Up	Up	Up
Hexokinase	NS	Down	Down	NS
Phosphoglycerate mutase	NS	Up	NS	Up
6-phosphofructokinase	Up	Up	Up	NS
Enolase	Up	NS	Up	NS
Phosphoglycerate kinase	Up	Up	Up	Up
Pyruvate kinase	Up	Up	NS	Up
Pyruvate dehydrogenase	Up	Up	Up	NS
Glyceraldehyde-3-phosphate dehydrogenase	Up	Up	Up	Up
Citrate cycle (TCA)				
Succinyl-CoA synthetase	NS	Down	Down	Down
Citrate synthase	NS	Up	Up	Up
Isocitrate dehydrogenase	NS	Up	Up	Up
Malate dehydrogenase	Up	NS	Up	Up
Aconitate hydratase	Down	Down	Down	Down
Succinate dehydrogenase	Up	Up	Up	Up
alpha-ketoglutarate dehydrogenase complex	NS	Up	NS	Up
Pentose phosphate pathway (PP)				
Transketolase	Up	Up	Up	NS
Ribose 5-phosphate isomerase A	Down	Down	Down	Down
6-Phosphogluconolactonase	Up	Up	Up	Up
Fatty acid degradation (FAD)				
Enoyl-CoA hydratase	Up	NS	Up	Up
Acyl-CoA dehydrogenase	Up	Up	-	Up
Fatty acid biosynthesis (FAB)				
Fatty acid synthase beta subunit	Down	Down	Down	Down
Fatty acid synthase alpha subunit Fatty acid synthase alpha subunit FasA	Down	NS	Down	Down
Almitoyl-protein thioesterase	Down	Down	NS	Down
Amino acids metabolism (AAM)				
Glycine hydroxymethyltransferase	NS	Up	Up	Down
Ketol-acid reductoisomerase	NS	Up	Up	Up
3-Isopropylmalate dehydratase	Down	Down	Down	Down
Chorismate mutase	Up	Up	Up	Up
NAD+ dependent glutamate dehydrogenase	NS	Down	Down	Down
Anthranilate phosphoribosyltransferase	Up	Up	NS	Up
O-acetylhomoserine	Up	Up	Up	Up
Aspartate aminotransferase	Up	Down	Down	Down
Fructose and mannose metabolism (FMM)				
Mannitol-1-phosphate 5-dehydrogenase	NS	Up	Down	Up
Mannitol 2-dehydrogenase	Up	Up	NS	NS
Starch and sucrose metabolism				
Phosphoglucomutase	NS	NS	Up	Up

Up and Down mean the metabolites up- and down-regulated in the gene transcriptional level; NS represented not significantly changed; “-” was not detected.

### Validation of transcriptome data by RT-qPCR

Expression profiles obtained from the RNA-seq analysis were validated by the selection of 10 contigs (C2.875, C6.493, C6.451, C5.27, C5.674, C4.214, C5.137, C5.136, C5.129, and C5.126) involved in the reproduction, pigment biosynthesis, and primary metabolism for RT-qPCR analysis. In every case, RT-qPCR data were consistent with the sequencing data (Supplementary Figure S4).

## Discussion

To reduce the cost of MP production, RB was used as a substrate for *M. purpureus* M9 fermentation. In this study, Man and Opti significantly increased pigment production, with the color values that were 18.69 fold and 7.53 fold higher than that of RB and reached up to 76.65 and 30.90% of the value of Rice, respectively. These nutritional ingredients transformed MP constituents as follows: Man notably improved the yields of two orange pigments, O1 and O2, which were 69.49 and 95.36% higher than that of Rice. Man exhibited effective effects on pigment production, suggesting that carbon source, especially Man, play an important role in pigment biosynthesis based on RB medium. Liu et al. (2020) reported that the yields of extracellular MP production fermented with rice straw hydrolysate in combination with glucose was 2.42 fold higher than that fermented with rice straw hydrolysate alone and 61.10% of that in submerged fermentation with glucose-based medium. The performance of Man in this study was superior to the results of report. Man could be converted back to fructose using mannitol 2-dehydrogenase and then fructose was phosphorylated to fructose-6-phosphate, which is an important intermedium of EMP. Furthermore, Man could turn into EMP through FMM. In addition, when Rice was used as a substrate, the maximum color value was obtained. Amylose and amylopectin, the main components of Rice, seemed to contribute to MP production. In fact, amylose and amylopectin are polysaccharides that can be decomposed into glucose-1-phosphate by amylase. Glucose-1-phosphate can transform to glucose-6-phosphate by phosphoglucomutase. Glucose-6-phosphate is also an important intermedium of EMP. Thus, amylose and amylopectin could turn into EMP through starch and sucrose metabolism. EMP could produce acetyl-CoA and malonyl-CoA, which are the precursors for MP biosynthesis. Meanwhile, the expression of phosphoglucomutase was upregulated in the Rice vs. RB group. Mannitol 2-dehydrogenase was upregulated in the Man vs. RB group. The gene expression of a series of enzymes (glucose-6-phosphate isomerase, phosphoglycerate mutase, etc.) involved in EMP was upregulated in the Man vs. RB and Rice vs. RB groups (Table 3). Therefore, sugar alcohols and rice starch could indirectly convert into the precursors for MP production.

Few studies are focused on the morphology and development of *Monascus* fermented with agricultural by-products. In this study, different nutritional ingredients remarkably influenced the growth and development of *M. purpureus* M9. Biomass and colony diameter of Opti presented the maximum value among different groups. The amount of cleistothecia of Opti and Man was significantly higher than the other groups, whereas AN and RB produced much more conidia compared to others. The results indicated that the synthesis of carbon source, nitrogen source and mineral ion sources is important for hyphal growth in *Monascus*.

Comparative transcriptome analysis was performed to explore the transcription levels of related genes involved in pigment biosynthesis, fungal growth, and spore development. In terms of pigment biosynthetic gene cluster, the expression levels of genes in the RB and AN group were low, while Opti, Man, and Rice showed high transcription level, which was in agreement with pigment production. With respect to reproduction, genes related to sexual development were upregulated in the Opti vs. RB, Man vs. RB, and Rice vs. RB groups. Meanwhile, genes linked to asexual sporulation were upregulated in the AN vs. RB group. This finding was consistent with the amount of sexual and asexual spores. To summarize, different nutritional ingredients led to changes in pigment production, asexual and sexual development, and growth of *M. purpureus* M9 through the regulation of related gene expression.

**Figure 6:**
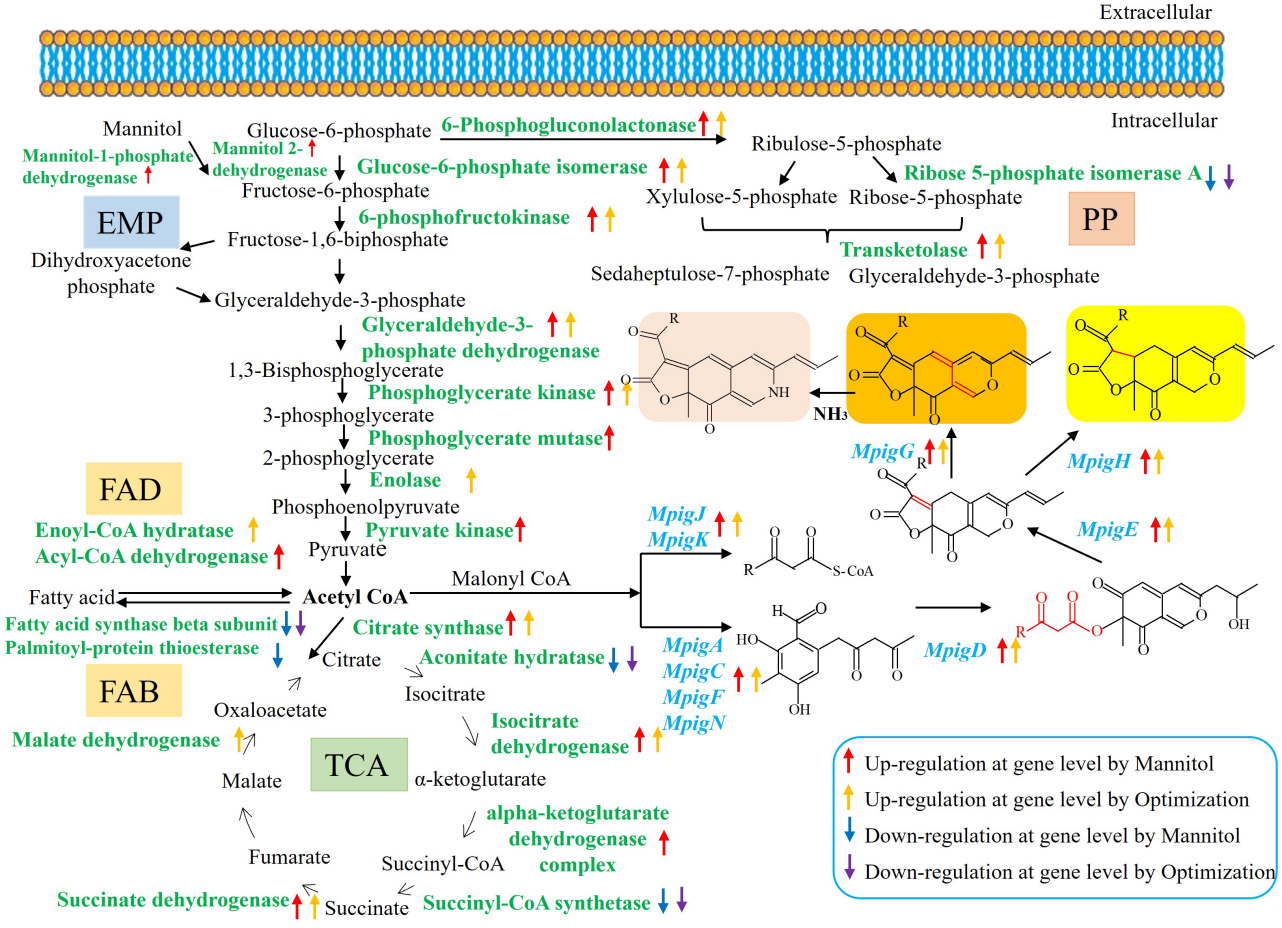
The regulation of the proposed models of Man and Opti of the primary metabolism for MP biosynthesis in *M. purpureus* M9.

KEGG pathway enrichment and DEG analysis indicated that the addition of nutritional ingredients affected the primary metabolism. As shown in Figure 1A, Man and Opti significantly increased MP production, therefore the primary and secondary metabolism of *M. purpureus* M9 cultivated with Man and Opti were analyzed. As shown in Figure 6, Man and Opti upregulated the gene expression of a series of enzymes involved in EMP and then generated more acetyl-CoA and malonyl-CoA for MP biosynthesis. There was a close association between EMP and PP pathway, thus gene expression associated with the PP pathway indirectly influenced acetyl-CoA concentration. The PP pathway also could provide NADPH for cell growth and metabolism. Man and Opti upregulated gene expression linked to the FAD pathway but downregulated gene expression related to the FAB pathway, which offered more substrates for MP biosynthesis. Acetyl-CoA is a product of FAD by beta-oxidation pathway. Acetyl-CoA participated in the TCA cycle, a core pathway for energy and substance metabolism in almost all organisms. In both the Man vs. RB and Opti vs. RB groups, the upregulation or downregulation of the TCA cycle could affect acetyl-CoA concentration. AAM not only provided the intermediates for the TCA cycle but also produced amine derivatives that constituted red MPs. In addition, Man could be phosphorylated to mannitol-1-phosphate and then transformed to fructose-6- phosphate by mannitol-1-phosphate 5-dehydrogenase. On the other hand, Man could turn to fructose by mannitol 2-dehydrogenase and then fructose could be converted to fructose-6- phosphate, which was an important intermediate of EMP (Supplementary Figure S5). Therefore, the Man metabolism has a close relationship with EMP. In summary, Man and Opti improved MP synthesis by regulating the primary metabolism, including EMP, PP pathway, TCA cycle, FAD, FAB, AAM, and FMM to provide the precursors (acetyl-CoA and malonyl-CoA) for MP biosynthesis.

In conclusion, this study first provided the parameters of nutritional ingredients to improve MP production with RB as a substrate. Then, the molecular mechanism of nutritional ingredients for enhancing MP biosynthesis was explained by comparative transcriptome analysis. These results will assist in the industrial production of MPs.

## Data availability statement

The datasets presented in this study can be found in online repositories. The names of the repository/repositories and accession number(s) can be found in the article/Supplementary material.

## Author contributions

DC: Data curation, Funding acquisition, Methodology, Writing – original draft, Writing – review & editing. HL: Investigation, Writing – original draft.

## Footnotes

^1^ https://www.ncbi.nlm.nih.gov/datasets/genome/GCA_003184285.1/
