# Supplementary material for: Mannitol improves Monascus pigment biosynthesis with rice bran as a substrate in Monascus purpureus
Source: Front Microbiol. 2023 Dec 13;14:1300461. doi: 10.3389/fmicb.2023.1300461 (PMC10753769; doi:10.3389/fmicb.2023.1300461)
Supplement: Supplementary file 1 [file Data_Sheet_1.docx]

Table S1. Primers for RT-qPCR analyzing the expression levels of genes

| primers | sequence |
| --- | --- |
| *β-actin*F | 5'-TTCGAGACCTTCAACGCCC-3' |
| *β-actin*R | 5'-ACCCTCGTAGATGGGAACGA-3' |
| C5.137F | 5'-TGTCCGACGAGTTTCTGCAA-3'  GACCCAGATCCCACCTACCT  -3’ |
| C5.137R | 5'-TATCAACGCTGCTTGGGCAT-3' |
| C5.136F | 5'-TCTGCAGTATGCCATGTGGG-3' |
| C5.136R | 5'-ATGGCACCGTCACTTAGCTC-3' |
| C2.875F | 5'- GTCGGCGAGAAGAGTGTTGA-3' |
| C2.875R | 5'- TGGTACCGTTGGAGAAAGGC-3' |
| C6.493F | 5'- GCTTCGCGATGACATGTTCC-3' |
| C6.493R | 5'- GCACCGAGTTGACTTCCTCA-3' |
| C6.451F | 5'- AAAGCATGGCCGATTGGAGA-3' |
| C6.451R | 5'- TGAGACTCTCCGCAACAACC-3' |
| C5.27F | 5’-CTCGTGTCACCTTCATGGCT-3' |
| C5.27R | 5'- CGACCGTTGGGACATGTGTA-3' |
| C5.674F | 5'- ATCATCTCCAGTTTGCCGGG-3' |
| C5.674R | 5'- ACACAGTTCTCATTGCCCCC-3' |
| C4.214F | 5'- TGGCGTCATCCTTGAGAACC-3' |
| C4.214R | 5'- TGTGTGGCGAAGTAGGCATT-3' |
| C5.129F | 5'-ACGAAACCCTCCATGACACC-3' |
| C5.129R | 5'-TGCAGACAGCCTTGTGGTAG-3' |
| C5.126F | 5'-TCCCATGGCCAGGCAATATC-3' |
| C5.126R | 5'-TGTGACGAGCTTTCCACCTC-3' |

Table S2 Quality parameters of filter reads of different nutritional ingredient samples

| Sample name | Clean read | Clean base | Q20 | Q30 |
| --- | --- | --- | --- | --- |
| ANA | 42.74 | 6.41 | 94.95 | 88.91 |
| ANB | 42.76 | 6.41 | 95.23 | 89.41 |
| ManA | 42.02 | 6.3 | 95.16 | 89.36 |
| ManB | 42.2 | 6.33 | 95.06 | 89.13 |
| OptiA | 42.61 | 6.39 | 94.81 | 88.62 |
| OptiB | 42.46 | 6.37 | 94.9 | 88.8 |
| RBA | 42.17 | 6.33 | 94.9 | 88.78 |
| RBB | 42.54 | 6.38 | 95 | 89.01 |
| RiceA | 42.57 | 6.38 | 94.87 | 88.72 |
| RiceB | 42.54 | 6.38 | 95.12 | 89.14 |

Table S3 Genome mapping ratio of different nutritional ingredient samples

| Sample name | Total mapping genome ratio | Uniquely mapping genome ratio |
| --- | --- | --- |
| ANA | 78.13 | 48.56 |
| ANB | 78.6 | 51.64 |
| ManA | 86.44 | 55.7 |
| ManB | 86.26 | 54.56 |
| OptiA | 87.67 | 54.46 |
| OptiB | 87.49 | 54.9 |
| RBA | 86.85 | 53.96 |
| RBB | 87.09 | 54.07 |
| RiceA | 86.54 | 52.56 |
| RiceB | 87.11 | 55.81 |


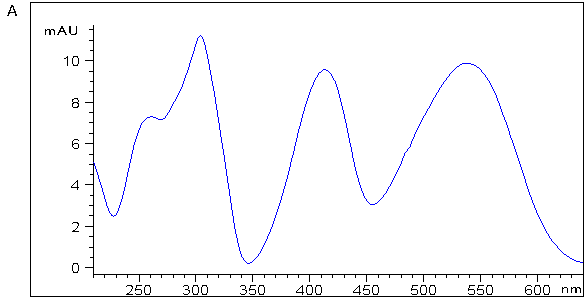

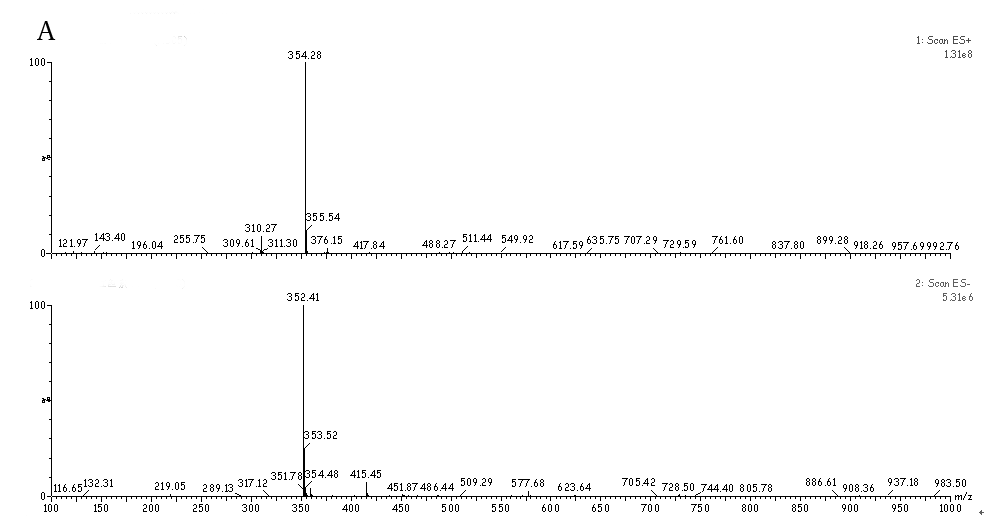


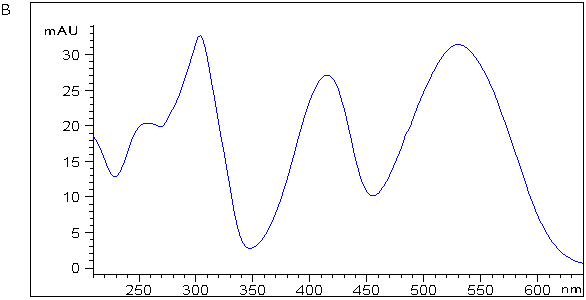

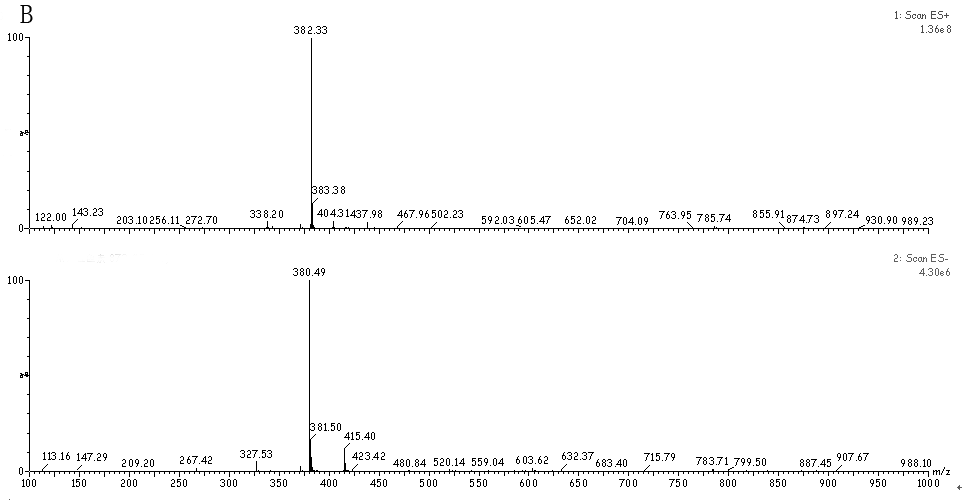


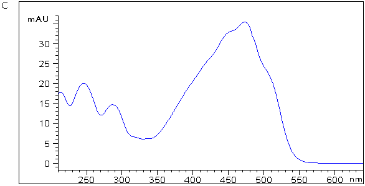

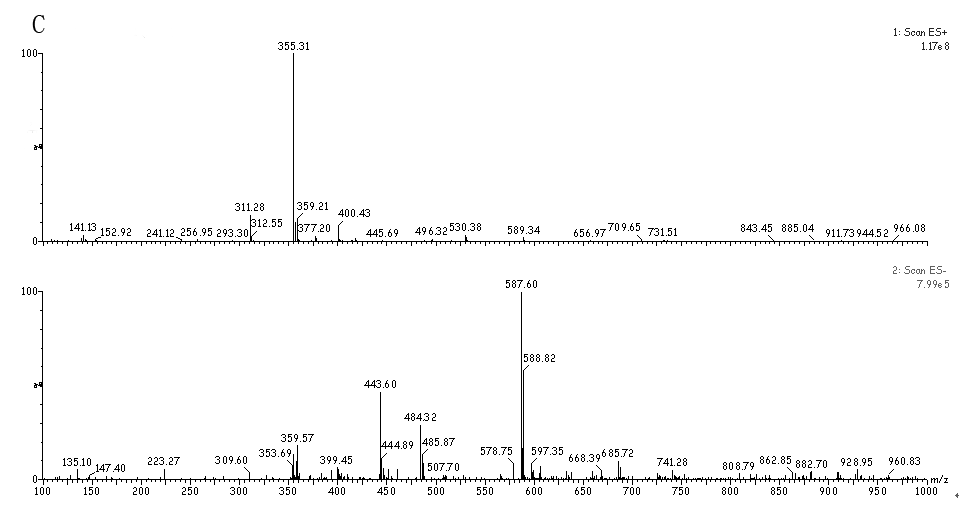


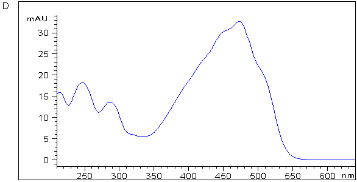

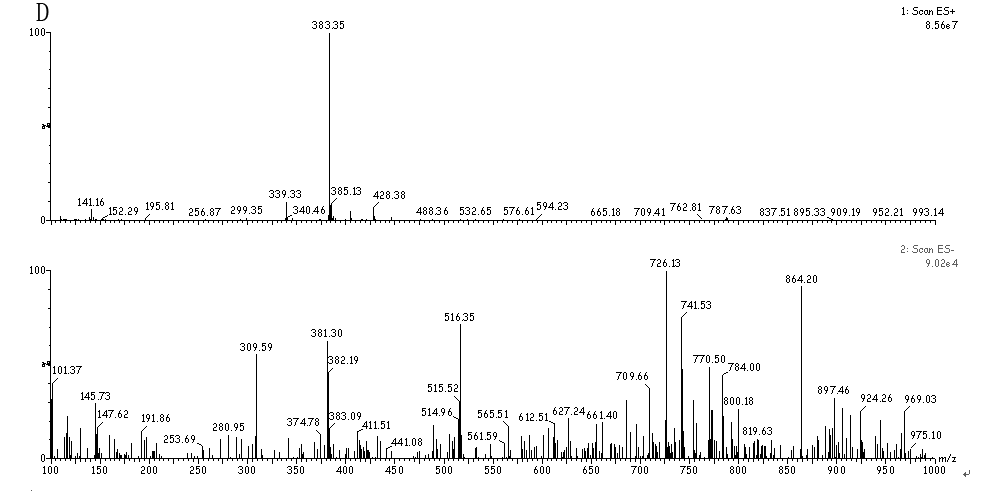


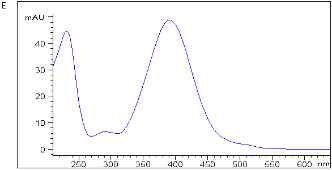

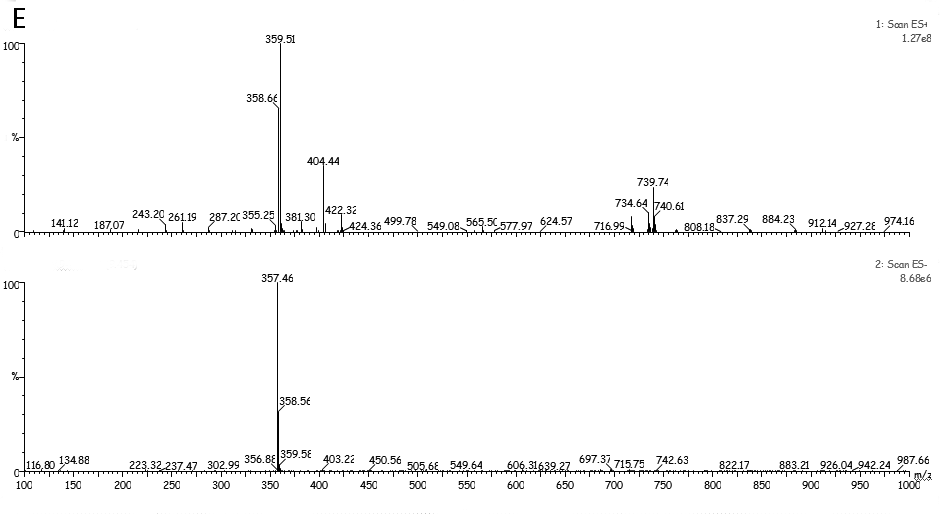


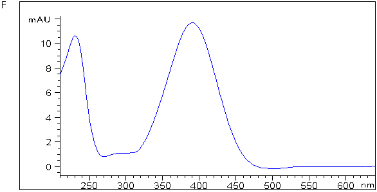

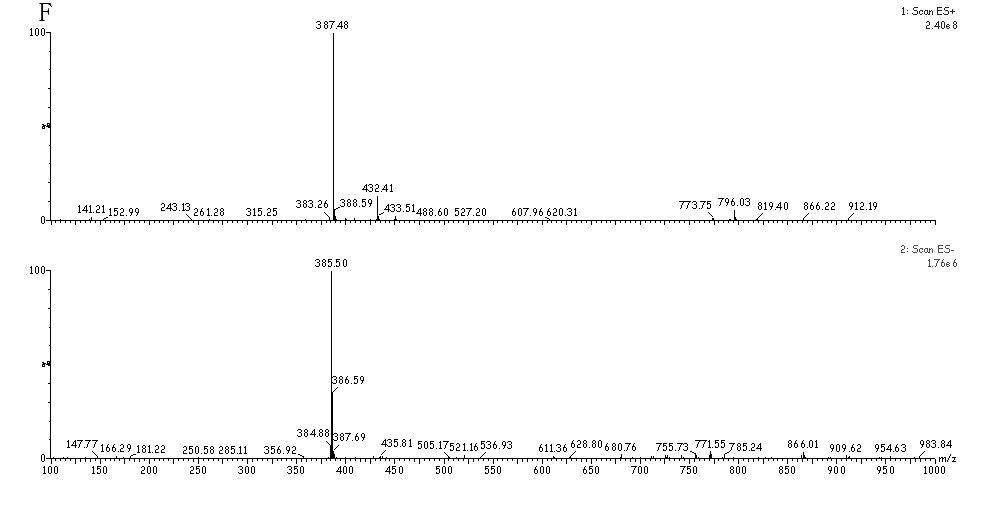


Figure. S1 The mass spectra and spectrum of Rubropunctatamine (a) and Monascorubramine (b), Rubropunctatin (c) and Monascorubrin (d), Monascin (e) and Ankaflavin (f).


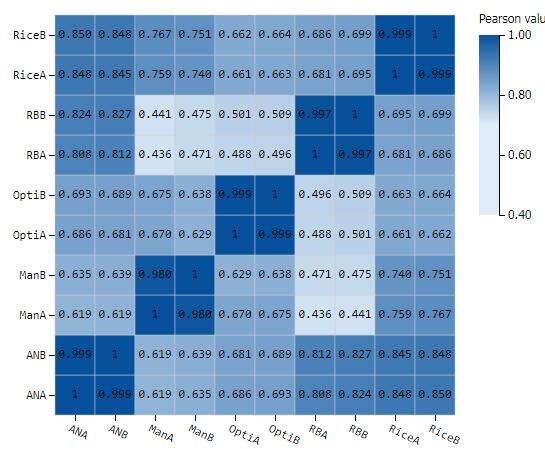


Figure. S2 Heat map of correlation analysis of different nutritional ingredient samples


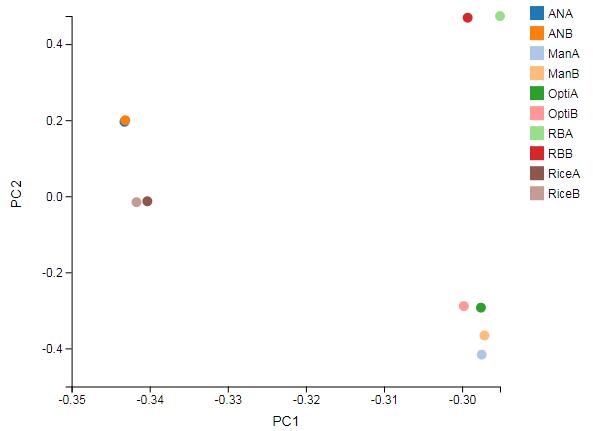


Figure. S3 Principal component analysis of different nutritional ingredient samples


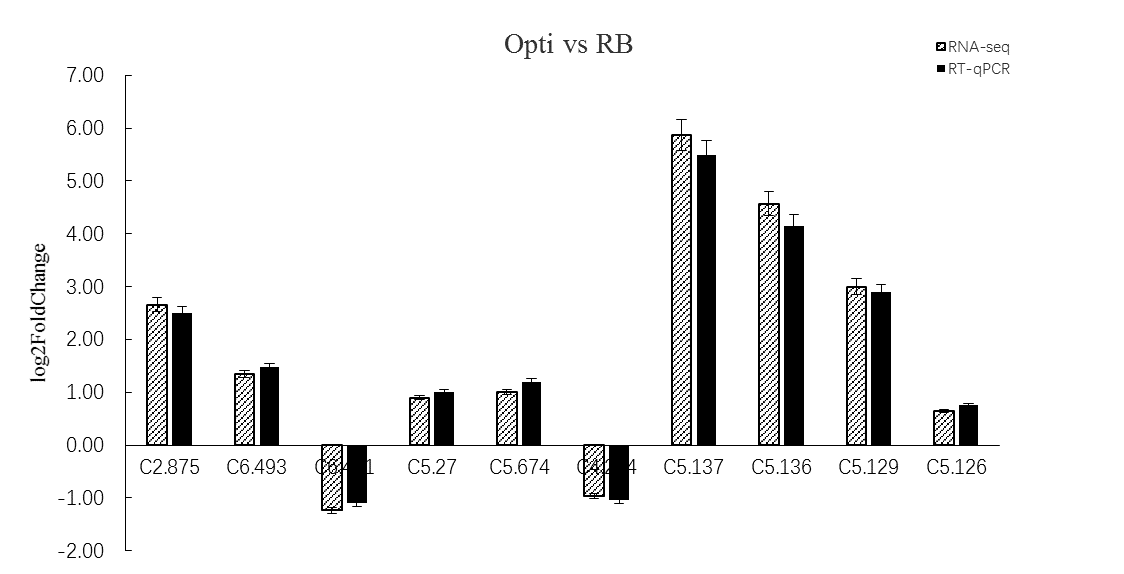


Figure. S4 Differences of the expression levels of genes between RT-qPCR and RNA-Seq


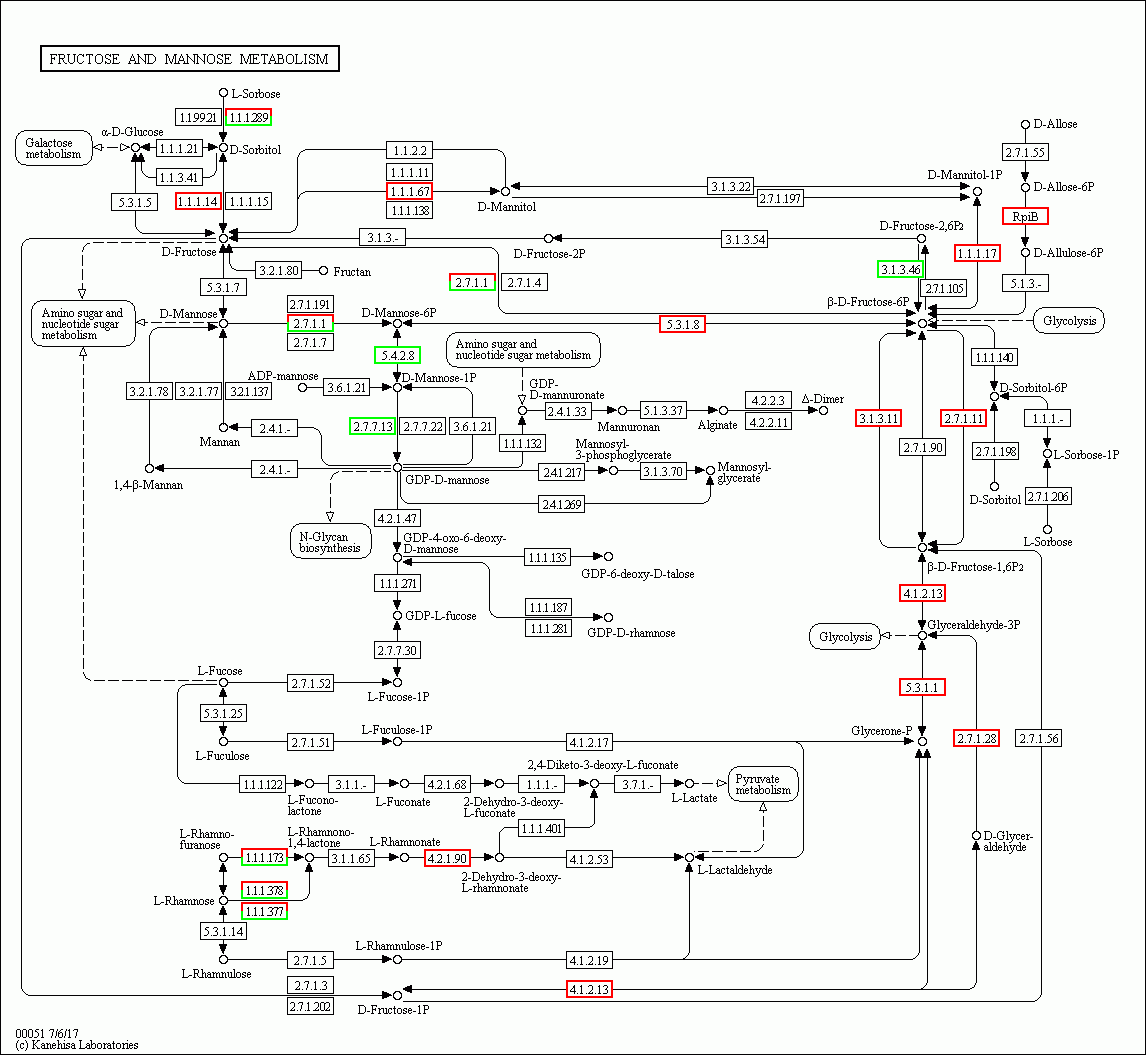


Figure. S5 Fructose and mannose metabolism generated by KEGG analysis in Man vs RB set. Red boxes indicate significantly increased expression. Green boxes indicate significantly decreased expression.
